# Farnesyl Diphosphate Synthase Gene Associated with Loss of Bone Mass Density and Alendronate Treatment Failure in Patients with Primary Osteoporosis

**DOI:** 10.3390/ijms25115623

**Published:** 2024-05-22

**Authors:** Werbson Lima Guaraná, Camilla Albertina Dantas Lima, Alexandre Domingues Barbosa, Sergio Crovella, Paula Sandrin-Garcia

**Affiliations:** 1 Keizo Asami Institute, Biosciences Center, Federal University of Pernambuco, Recife Campus, Recife 50670-901, Brazil; camilla.lima@ufpe.br; 2 Department of Oceanography, Technology and Geoscience Center, Federal University of Pernambuco, Recife Campus, Recife 50740-550, Brazil; 3 Rheumatology Division, Clinical Hospital of Federal University of Pernambuco, Recife Campus, Recife 50740-900, Brazil; domingues.reumato@gmail.com; 4 Laboratory of Animal Research Center (LARC), Qatar University, Doha P.O. Box 2713, Qatar; 5 Department of Genetics, Biosciences Center, Federal University of Pernambuco, Recife Campus, Recife 50730-120, Brazil

**Keywords:** FDPS, genetic variant, postmenopausal osteoporosis, pharmacogenetic, alendronate sodium, aminobisphosphonates

## Abstract

Aminobisphosphonates (NBPs) are the first-choice medication for osteoporosis (OP); NBP treatment aims at increasing bone mineral density (BMD) by inhibiting the activity of farnesyl diphosphate synthase (FDPS) enzyme in osteoclasts. Despite its efficacy, inadequate response to the drug and side effects have been reported. The A allele of the rs2297480 (A > C) SNP, found in the regulatory region of the *FDPS* gene, is associated with reduced gene transcription. This study evaluates the *FDPS* variant rs2297480 (A > C) association with OP patients’ response to alendronate sodium treatment. A total of 304 OP patients and 112 controls were enrolled; patients treated with alendronate sodium for two years were classified, according to BMD variations at specific regions (lumbar spine (L1-L4), femoral neck (FN) and total hip (TH), as responders (OP-R) (*n* = 20) and non-responders (OP-NR) (*n* = 40). We observed an association of CC genotype with treatment failure (*p* = 0.045), followed by a BMD decrease in the regions L1-L4 (CC = −2.21% ± 2.56; *p* = 0.026) and TH (CC = −2.06% ± 1.84; *p* = 0.015) after two years of alendronate sodium treatment. Relative expression of the *FDPS* gene was also evaluated in OP-R and OP-NR patients. Higher expression of the *FDPS* gene was also observed in OP-NR group (FC = 1.84 ± 0.77; *p* = 0.006) when compared to OP-R. In conclusion, the influence observed of *FDPS* expression and the rs2897480 variant on alendronate treatment highlights the importance of a genetic approach to improve the efficacy of treatment for primary osteoporosis.

## 1. Introduction

Osteoporosis (OP) is a degenerative osteometabolic disease characterized by progressive loss of bone mineral density (BMD) and deterioration of bone microarchitecture, which lead to an increase in skeletal fragility and, consequently, greater susceptibility to fractures [1]. It is estimated that around 20 to 30% of patients die because of fractures or develop secondary diseases and morbidities. Notably, those who survive may not fully recover after the fractures [2]. In addition, about half of the fractures diagnosed in OP patients are also related to unspecific or inefficient drug therapies, increasing morbidity and mortality rates during the first year of treatment [3,4].

The aminobisphosphonates (NBPs) are a class of antiresorptive drugs widely used as the first choice for OP treatment in postmenopausal women, as they prevent bone resorption, with an increase of BMD and recovery to normal levels of biochemical markers of bone turnover [5,6,7]. Once absorbed, the NBPs act directly on the mineralized bone, causing osteoclast apoptosis via blockage of the mevalonate pathway, specifically acting on the enzyme farnesyl diphosphate synthase (FDPS) [6,8]. Nevertheless, despite the recognized effectiveness in reducing osteoporotic fractures, a considerable proportion of patients do not respond to NBP therapy, presenting significant loss of BMD due to non-suppression of bone resorption [9]. The prevalence of inadequate response to antiresorptive drugs is largely discordant among different studies and varies between 9.5 and 53% [10].

Several studies have demonstrated the multifactorial etiology of OP and fracture risk susceptibility, showing a significant influence of genetic factors in bone loss acceleration [11,12,13,14]. Furthermore, with the elucidation of the mevalonate pathway’s role as the target of the NBPs class, in particular, the enzyme FDPS, genetic variations occurring in the *FDPS* encoding gene may have an important role both in therapy effectiveness and the occurrence of side effects, influencing the response or resistance to NBP treatment [15,16]. Thus, the knowledge of genes associated with the response to NBPs is a challenging and highly promising issue aimed at providing individual personalized therapy in osteoporosis patients [17].

In postmenopausal OP, several single nucleotide variants (SNVs) in different genes have been associated with BMD change [17,18]. In this context, the rs2297480 (A > C) variant in the *FDPS* gene could be considered an informative one since FDPS is a crucial enzyme of the mevalonate pathway and the target of several NBPs [11,12,14,19]. Furthermore, the *FDPS* rs2297480 is localized at the 5’UTR region of the gene, 778 bp upstream of the translation site. In silico analysis had predicted that allele A could create a bidding site for the hematopoiesis transcription factor Runx1, leading to a decrease in *FDPS* gene transcription [11,12,13]. Thus, this variant can impact the *FDPS* mRNA translation level, affecting the effectiveness of NBP therapy [14].

In previous studies, the *FDPS* rs2297480 variant was associated with changes in the BMD of osteoporosis patients treated with bisphosphonates, where the AA genotype was associated with BMD gain while the CC with loss of BMD during treatment [12,13,20]. Furthermore, pharmacogenetics meta-analysis identified this genetic variant as a potential biomarker for response to bisphosphonate therapy [21]

Therefore, the present study evaluated the association and correlation of the SNV rs2297480 with OP susceptibility, biochemical markers, and response to alendronate therapy. Furthermore, to assess the impact of the variant on the mRNA expression of the *FDPS* gene, we analyzed its expression profile in relation to the therapeutic failure of alendronate sodium among a population-based sample of postmenopausal women with osteoporosis.

## 2. Results

### 2.1. Comparison between OP Patients and Controls

#### 2.1.1. Clinical Characterization and Biochemical Marker Levels

A total of 416 patients were included in the study according to inclusion criteria, of which 304 had a diagnosis of OP and were compared to 112 controls. In the clinical analysis, no statistically significant differences were observed in age, the onset of menarche, and the age at menopause. Concerning BMD means, the OP group presented lower density at all measured regions (L1-L4, TH, and FN) (*p*-value < 0.001). Additionally, when considering biochemical markers levels, the OP group showed lower levels of 25-hydroxyvitamin D (29.09 ± 9.24 ng/mL) when compared to the control group (32.26 ± 11.03 ng/mL) (*p*-value = 0.046). No significant difference was observed between the following: calcium (OP: 9.52 ± 1.20 mg/dL; control: 9.52 ± 0.87 mg/dL; *p* = 0.622) and ALP (OP: 82.20 ± 48.48 U/L; control: 90.69 ± 62.86 U/L; *p* = 0.499) and gamma-glutamyltransferase (GMT) (OP: 22.45 ± 7.44 U/L; control: 22.22 ± 7.36 U/L; *p* = 0.829) and phosphorus (OP: 3.50 ± 0.67 mg/dL; control: 3.59 ± 0.56 mg/dL; *p* = 0.353) and PTH (OP: 54.48 ± 27.07 pg/mL; control: 59.13 ± 45.72 pg/mL; *p* = 0.287) and creatinine clearance (OP: 104.00 ± 11.67 mL/min; control: 106.10 ± 10.81, *p* = 0.196) and magnesium (OP: 2.06 ± 0.28 mg/dL; control: 2.02 ± 0.47 mg/dL; *p* = 0.956) (Table 1).

#### 2.1.2. rs2297480 *FDPS* SNV and Susceptibility to Developing OP

The *FDPS* (rs2297480) minor allele C allelic frequencies were 33% and 29% in OP and control, respectively. A borderline statistical association was observed in the recessive model for OP susceptibility (OR = 2.14; *p* = 0.05). No significant association was observed for the codominant and dominant models tested (*p* = 0.15 and *p* = 0.79, respectively). There was no evidence of deviation from Hardy–Weinberg equilibrium (Table 2).

#### 2.1.3. rs2297480 *FDPS* SNV and Correlation with Biochemical Markers

Concerning the analysis of the association between the means of the levels of biochemical markers and the genotypes for SNV *FDPS* rs2297480, a significant increase in the PTH serum level (AA × AC × CC; *p* < 0.001) was observed between patients with CC genotype (76.45 ± 31.57 pg/mL) and the genotypes AA (48.34 ± 23.42 pg/mL) and AC (62.20 ± 39.18 pg/mL). A significant difference in ALP serum level was also observed between the AA and CC genotypes (*p* = 0.020), with the CC genotype group presenting higher ALP serum levels compared with the AA genotype (AA = 82.22 ± 46.51; CC = 96.70 ± 42.11 U/L) (Table 3).

### 2.2. Comparison between Responder and Non-Responder Patients

#### 2.2.1. rs2297480 *FDPS* SNV and Correlation with Treatment Response

A total of 60 OP patients, 40 OP-NR, and 20 OP-R were enrolled for treatment response analysis. No statistically significant difference was observed in age mean, age at menarche, and menopause. The magnesium level showed a statistically significant lower concentration (1.76 ± 0.25 mg/dL) in the OP-R group compared to the OP-NR group (2.1 ± 0.20 mg/dL) (*p*-value = 0.028). No statistically significant difference was observed among the other biochemical markers analyzed (Table 4).

Genotype analysis showed, for the recessive model, that the CC genotype was significantly associated with the non-responder OP (*p* = 0.045), corroborating the borderline result for the study of OP association. However, only the OP-NR group showed homozygosity to the CC genotype (n = 5). No significant difference was observed in the C allele or genotypes for other frequencies between OP-NR (36%) and OP-R (33%) (*p* = 0.83) (Table 5).

#### 2.2.2. rs2297480 *FDPS* SNV and Correlation with BMD

**Figure 1:**
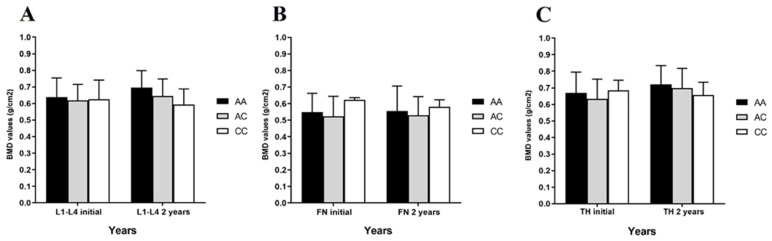
Average of bone mineral densities (BMDs) (g/cm^2^) before and after 2-year follow-up of alendronate sodium treatment across rs2297480 genotypes from: (**A**) lumbar spine (L1-L4), (**B**) femoral neck (FN) and (**C**) total hip (TH). Data are expressed as the mean ± standard deviation. No significant difference was observed between BMD means according to the *FDPS* rs2297480 AA, AC, and CC genotypes after 2 years of alendronate treatment.

The average BMD levels (g/cm^2^) were measured via dual-energy X-ray absorptiometry (DXA) exam after two years (vertebrae L1-L4, femoral neck, and total hip) and compared with the *FDPS* rs2297480 genotypes (Figure 1).

No significant difference was observed between baseline BMD means and average BMD measures taken 2 years later: LN (AA × AC × CC *p* = 0.295); FN (AA × AC × CC *p* = 0.391); and TF (AA × AC × CC *p* = 0.345).

**Figure 2:**
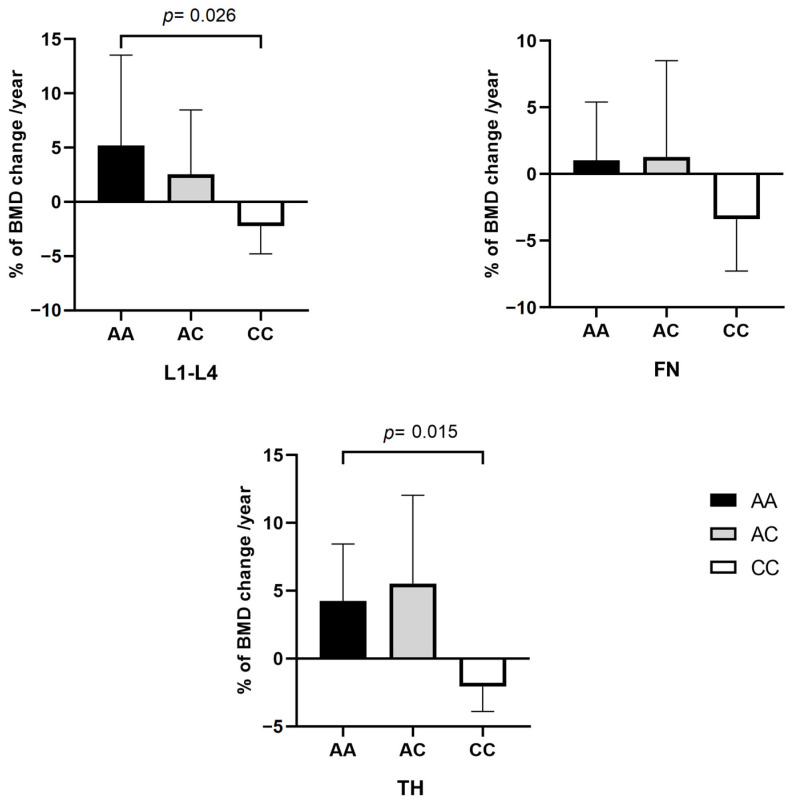
Changes in bone mineral density (BMD) from lumbar (L1-L4), femoral neck (FN), and total hip (TH) after alendronate therapy compared between *FDPS* rs2297480 genotypes. Data are expressed as average annual percentage change of BMD compared to baseline BMD after two years of alendronate treatment. In the L1-L4 and TH regions, the genotypes AA and CC showed significant differences (*p* < 0.05).

The annual average percentage change of BMD for both AA and AC genotypes presented an increase during the two-year follow-up. On the other hand, the CC genotype was associated with loss of BMD in all measured regions with statistically significant on the lumbar (L1-L4) region (AA = 5.21% ± 8.33; AC = 2.54% ± 5.92; CC = −2.21% ± 2.56; *p* = 0.026), and total hip (TH) (AA = 4.25% ± 4.18; AC = 5.53% ± 6.49; CC = −2.06% ± 1.84; *p* = 0.015). Although similar results among the genotypes, the average of annual change on the femoral neck (FN) was observed, they did not reach the statistical significance (AA = 1.03% ± 4.37; AC = 1.28% ± 7.22; CC = −3.39% ± 3.88; *p* = 0.066) (Figure 2).

### 2.3. Expression Analysis of FDPS in Responder and Non-Responder OP Patients

**Figure 3:**
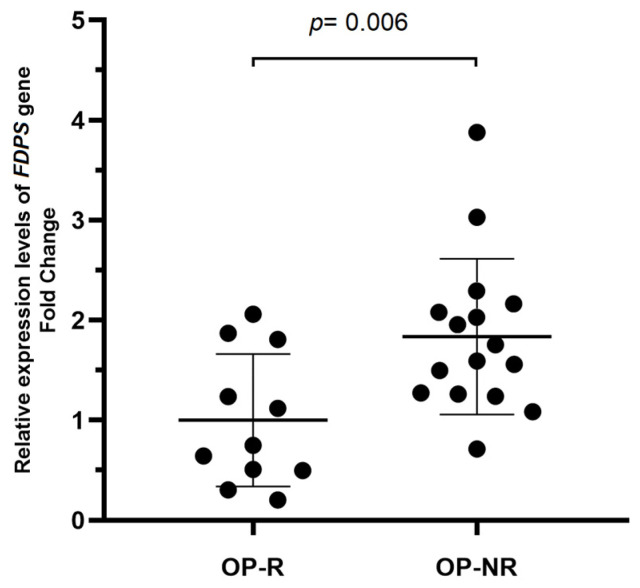
*FDPS* gene expression level of OP treatment non-responder patients (OP-NR) in relation to responder patients (OP-R). The relative expression of *FDPS* is shown in scatter plots. OP-NR patients (fold change = 1.84) compared to OP-N patients (fold change = 1). Individuals are presented as black circles. Data are expressed as mean (bold line) and ± standard deviation (thin line).

A total of 20 randomly selected OP-NR and 20 OP-R patients were evaluated for *FDPS* gene expression. When we compared the *FDPS* gene expression level between OP-NR and OP-R, a 1.84-Fold Change (FC) increase was detected in the non-responder group when compared to the responder group (FC = 1.84 ± 0.77 *p* = 0.006) (Figure 3).

#### 2.3.1. *FDPS* Gene Expression and Biochemical Marker Correlation

A correlation analysis was performed between the *FDPS* gene expression and the serum levels of biochemical bone markers (25-hydroxyvitamin D, calcium, FA, PTH, phosphorus, and magnesium).

In the responder group, we observed a negative correlation between serum phosphorus levels and *FDPS* gene expression (Pearson = −0.6954; 95% CI = −0.9140–−0.1636; *p* = 0.017). Furthermore, a positive correlation was observed between serum magnesium levels (Pearson = 0.9262; 95% CI = 0.2399–0.9952; *p* = 0.023). The non-responder patients did not show a significant correlation of *FDPS* gene expression with any of the bone biochemical markers (Table 6).

#### 2.3.2. *FDPS* Gene Expression and SNV rs2297480 Genotypes 

**Figure 4:**
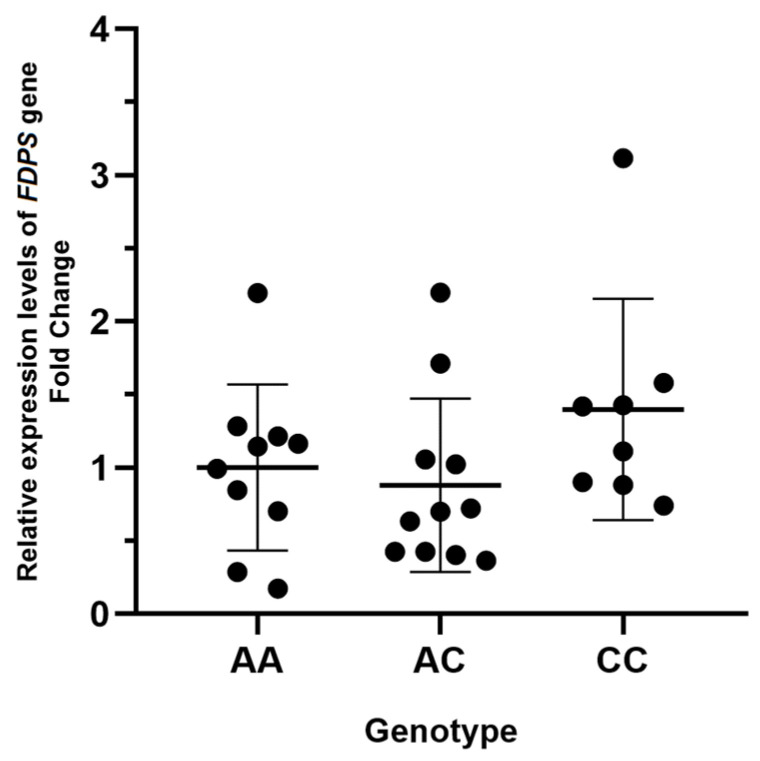
Genotype-guided expression analysis of *FDPS* gene. Genotype-guided relative expression of *FDPS* is shown in scatter plots. AC genotype (fold change = −1.14) and CC (fold change = 1.39) are compared to AA genotype. Individuals are presented as black circles. Data are expressed as mean (bold line) and ± standard deviation (thin line).

The genotype-guided expression analysis of the *FDPS* rs2297480 was performed, and the CC genotype presented with higher FC values. However, no statistically significant result was observed among the genotypes evaluated in comparison to AA patients: AC (FC = −1.14, *p* = 0.456) and CC (FC = 1.39; *p* = 0.221) (Figure 4).

## 3. Discussion

This study demonstrates a difference in response to alendronate sodium treatment among osteoporosis (OP) patients based on their *FDPS* genotypes. Our analysis revealed a significant correlation between the *FDPS* rs2297480 CC genotype and treatment non-responsiveness (*p* = 0.045), indicating a potential link between this genetic variation and therapeutic outcomes. Additionally, we observed a borderline statistical association between the CC genotype and an increased susceptibility to osteoporosis (*p* = 0.05). Furthermore, individuals with the CC genotype displayed higher serum levels of alkaline phosphatase (*p* = 0.020) and parathyroid hormone (PTH) (*p* < 0.001) compared to those with AA and AC genotypes. PTH contributes to bone remodeling by activating and recruiting osteoclasts. The increase in alkaline phosphatase levels is a response to bone degradation by osteoclasts, which is subsequently rebuilt by osteoblasts. [22,23]. Thus, according to the above-cited studies, the association between the CC genotype with higher PTH and alkaline phosphatase levels may be related to increased osteoclast activity due to an inadequate response of patients to alendronate treatment.

After two years of treatment, bone mineral density (BMD) measurements revealed that patients with the CC genotype experienced a decrease in BMD across all measured areas (L1-L4, femoral neck (FN), and total femur (TF)). Also, the annualized percentage change in BMD indicated a significant difference between those with AA and AC genotypes and those with the CC genotype. Specifically, individuals with the CC genotype showed a reduction in BMD (L1-L4 = −2.21% ± 2.56; TF = −2.06% ± 1.84), while those with AA and AC genotypes exhibited an increase in BMD in the lumbar spine (L1-L4) (AA = 5.20% ± 8.33; AC = 2.54% ± 5.92) and femoral regions (TF) (AA = 4.25% ± 4.18; AC = 5.53% ± 6.49). These findings suggest that patients with the CC genotype may have higher bone remodeling activity, leading to a failure in response to alendronate sodium treatment, which aligns with the elevated levels of alkaline phosphatase and PTH observed in this group. These results are concordant with the findings of Marini and colleagues [12], who evaluated the same variant in 234 Danish women with primary postmenopausal OP. The authors observed that patients with the CC genotype presented a lower increase in BMD than individuals carrying the AA and AC genotypes, who had similar BMD increases. In addition, a significantly lower decrease of Cross-laps bone turnover marker (CTX) was observed in the CC genotype compared to AA and AC *(p* = 0.049) after two years of treatment with NBPs. In another study, Olmos and colleagues [13] evaluated 191 women with postmenopausal OP and observed an association between rs2297480 polymorphism with the rate of increase of 1% per year in BMD in patients with AA genotype and a loss of 1.6% of BMD in patients with CC genotype, in an average period of 2.5 years of treatment with alendronate or risedronate (*p* = 0.001).

Concerning the expression profile of the *FDPS* gene, we observed that OP-NR group patients showed higher levels (FC = 1.84; *p* = 0.006) compared to the OP-R group. Since the FDPS enzyme is the main target of NBPS, changes in the gene expression may impact the protein level and the effectiveness of therapy. This relation was assessed in two studies conducted by Sugden and colleagues in 2005 [24] and Grove and colleagues in 2010 [25], using a mutant *Dictyostelium discoideum* amoeba (MR102) that overexpresses *FDPS*. Since alendronate functions as a cytotoxic drug targeting amoeba, the role of FDPS as a target protein becomes crucial when the gene is overexpressed. An increased amount of the target protein can shield the cell from the drug’s cytotoxic effects, necessitating a higher drug concentration to completely inhibit the protein. Consequently, elevated production of the FDPS protein results in increased resistance to NBP therapy. Our study’s findings align with this mechanism, as a higher expression of *FDPS* was noted in the osteoporosis non-responder (OP-NR) group. This increased expression may contribute to their heightened resistance to alendronate treatment, which is further supported by the smaller gains in bone mineral density (BMD) observed in this group after two years of treatment.

In the analysis of *FDPS* gene expression relative to the rs2297480, shown in Figure 4, patients with CC genotype presented higher *FDPS* expression (FC = 1.39) than AA and AC. According to Levy and colleagues [11] and Marini and colleagues [12], the *FDPS* allele “A” leads to a decrease in *FDPS* gene transcription; therefore, the results follow the expectation that the presence of a variant polymorphism could impact gene expression. Furthermore, a recent study by Marozik and colleagues [14] highlighted the influence of allelic combinations, increasing negative responses to bisphosphonate therapy. The study suggested that the combination of *FDPS* alleles dramatically increases the risk of negative response in patients with NBP treatment for postmenopausal OP, emphasizing the importance of identified allelic combinations of gene variants to evaluate the individual resistance or sensitivity to NBP treatment for the disease. Therefore, the lack of statistical association between rs229748 and *FDPS* expression level in this study must be considered carefully and elucidated in further studies considering the allelic combination. In addition, the small number of genotypes in this analysis also can be a limited factor for the study.

## 4. Methodology 

### 4.1. Subjects

For the association study, we randomly included 416 postmenopausal women, classified into two clinical groups according to the World Health Organization criteria based on the T-score of the BMD (g/cm^2^) measurement at lumbar spine (L1-L4) (LS), total hip (TH), and femoral neck (FN) using dual-energy X-ray absorptiometry (DXA)**.** The equipment calibration was daily performed using a standard spine phantom provided by the manufacturer. The patients were divided into two groups: (1) patients with OP (n = 304, average age 65.6 ± 7.1 years) and (2) control (n = 112, average age 61.2 ± 5.2 years). All the participants were from the State of Pernambuco and attended the Rheumatology Clinic of Hospital das Clínicas, Universidade Federal de Pernambuco (HC-UFPE). We calculated the annualized percent BMD change for each participant as follows: [(BMD2 − BMD1)/BMD1] × 100/time (in years) between assessments [13].

The OP patient’s inclusion criteria were postmenopausal women diagnosed with OP, with a follow-up of at least two years of treatment with alendronate sodium for at least 12 months according to the clinical guidelines in health supplements from the National Agency of Health of Brazil [26], using recommended 70 mg/week oral doses of alendronate sodium. All women were supplemented with 600 mg/day of elemental calcium and 400 IU/day of 25-hydroxyvitamin D_3_. The exclusion criteria were the presence of osteopenia, cancer, and the use of other drugs for bone disorders, such as anabolic agents, hormone replacement therapy, or selective estrogen receptor modulators at any moment of the treatment period.

Following the World Health Organization criteria, the control group included postmenopausal women without a diagnosis of OP or osteopenia, according to the DXA exam, and without fracture history after menopause. Individuals with cancer, diabetes, and other rheumatology diseases such as degenerative osteoarthritis of the spine and hip were excluded [27].

The analysis of therapeutic failure to alendronate sodium was carried out by an observational case-control study comparing two sample groups in treatment for at least two years: (1) patients with a decrease or no stabilization of BMD were classified as non-responders (OP-NR) (n = 20) and (2) patients who had stabilization or increase of BMD were classified as osteoporosis responder (OP-R) (n = 40) [28]. BMD was measured on the same machine at baseline and during 24 months of treatment. The response to alendronate therapy was evaluated according to the BMD trend in the LS and TH regions. The least significant change (LSC) was considered to determine the response to therapy (LS = 0.051 g/cm^2^; TH = 0.033 g/cm^2^). An increase in LS BMD value that exceeds the LSC was classified as a response to therapy, and a decrease was considered a non-responder. Patients with a change of LS BMD that did not exceed the LSC were not included in further analysis.

The clinical and biochemical bone markers levels were evaluated for patients and control group: age at menarche (years), age at menopause (years), 25-hydroxyvitamin D (ng/mL), calcium (mg/dL), alkaline phosphatase (ALP; U/L), gamma-glutamyltransferase (GMT; U/L), phosphorus (mg/dL), parathyroid hormone (PTH; pg/mL), creatinine clearance (mL/min) and magnesium (mg/dL).

The sample size was determined using G Power 3.1.9.7 software. Based on prior research [12,13,21], we employed a theoretical medium effect size (0.50 for the *t*-test; 0.3 for the Chi-square analysis; 0.3 for the correlation analysis; and 0.25 for the ANOVA test). The significance level (α) was set at 0.05, with a desired power of 0.8. The parameters utilized adhere to previous publications in G Power software studies [29,30,31].

All the participants provided written informed consent approved by the local Research Ethics Committee of the Center for Health Sciences, Federal University of Pernambuco (CEP/CCS/UFPE nº 513/11) following the rules of the 1964 Helsinki Declaration.

### 4.2. Genotyping

Genomic DNA extraction was performed from peripheral blood leukocytes using the rapid salting-out method [32]. Samples were genotyped for *FDPS* rs2297480 (C___2737970_10) with specific fluorogenic probes (Applied Biosystems, Foster City, CA, USA). All the experiments were performed on the Real-Time PCR ABI 7500 detection system (Thermo Fisher, Madison, WI, USA). 

### 4.3. Expression Analysis

The RNA was extracted from the peripheral blood of 40 patients of the OP-NR and OP-R group by TRIzol^®^ (Invitrogen, Carlsbad, CA, USA), according to the manufacturer’s instructions. The cDNA synthesis was performed from each RNA sample (input of 500 ng) using GoScript™ Reverse Transcription System (Promega, Madison, WI, USA) following the manufacturer’s instructions. *FDPS* gene expression assay was performed with a TaqMan^®^ gene-specific probe according to the manufacturer’s instructions (Assay ID Hs01578769_g1, Thermo Fisher, USA). For normalization, the following were used: the specific housekeeping gene primers *GAPDH* (Forward/Reverse: TGATGCCCCCATGTTCGT/GCAGGAGGCATTGCTGATGA) and *RPLP0* (Forward/Reverse: GCGACCTGGAAGTCCAACTA/TCTGCTTGGAGCCCACATTG, designed in NCBI Primer-BLAST (www.ncbi.nlm.nih.gov/tools/primer-blast/, accessed on 17 May 2024). The efficiency value for each primer was determined using slopes of standard curves from five 10-fold serial dilution points for each cDNA sample, starting with 5 ng of cDNA. The acceptable values were defined between 95% and 105%. For *FDPS* TaqMan^®^ probes, we considered a reaction efficiency of 100% ensured by manufacturers’ information [33]. For this, qPCR reaction was used: 5 ng of cDNA, 10 μM of each primer, 5 μL SYBR Green Master Mix (1×) (Thermo Fisher Scientific, USA), and ultrapure water to a final volume of 10 μL. The experiments were performed on Real-Time PCR with an ABI 7500 detection system (Applied Biosystems, Foster City, CA, USA). Relative quantitative expressions were calculated following the fold change (FC) method suggested by Schmittgen and Livak [34] for the assays: FC = 2^−ΔΔCq^ or FC = 2^ − [(Cq gene of interest − Cq internal control) sample A − (Cq gene of interest − Cq internal control) sample B].

### 4.4. Data Analysis

Statistical analysis for allelic and genotypic frequencies and Hardy–Weinberg equilibrium was performed using the SNPStats tool available at: https://www.snpstats.net/start.htm (accessed on 28 August 2023). Comparisons among the genotypes related to BMD and biochemical marker levels were performed using correlation analyses (Pearson) and variance (ANOVA) or Kruskal–Wallis tests as appropriate. When a significant difference was observed, the Tukey test was applied for pairwise comparisons of genotypes. Differences between groups were analyzed using Student’s T and ANOVA one-way tests. All statistical analyses were conducted using GraphPad Prism 8.0 (GraphPad Software, La Jolla, CA, USA). Differences were accepted significantly at *p*-values < 0.05. The power of the study was verified using G*Power software 3.1.9.2. 

## 5. Conclusions

In our study, we explored the genetic variant rs2297480 (A > C) in the *FDPS* gene as a potential biomarker for predicting the response to alendronate sodium treatment in postmenopausal women with OP. Our findings underscore the significant association of the CC genotype with decreased BMD and non-responsiveness to treatment. Furthermore, the higher *FDPS* gene expression observed in the OP-NR group also suggests a gene influence on the drug’s resistance. Despite these insights, the study is not without limitations. One significant limitation of this research is the focus on a single genetic variant, rs2297480, in the *FDPS* gene. While this SNP showed a strong correlation with treatment outcomes, osteoporosis is a multifactorial disease influenced by multiple genetic and environmental factors. Therefore, relying solely on this variant may overlook other genetic contributions that affect the disease process and treatment response. Despite this limitation, the study highlights the importance of simple and scalable genetic biomarkers like rs2297480 in clinical practice. As demonstrated, this genetic marker can potentially guide therapeutic decisions, particularly in determining the suitability of alendronate sodium treatment for individual patients. The integration of genetic testing into routine clinical practice could enhance treatment outcomes by aligning patients with the most effective therapies based on their genetic makeup. To improve the applicability of our findings, further research should focus on validating these results in larger, more diverse populations. In conclusion, while our study presents promising data on the utility of *FDPS* gene expression and the rs2297480 variant as a biomarker for alendronate sodium treatment response, the reliance on a single genetic variant is a notable limitation. Expanding genetic analysis in future research will be crucial for developing a more accurate and clinically applicable genetic profiling strategy in osteoporosis treatment. 

## Figures and Tables

**Table 1:** Clinical and biochemical markers of study patients.

Variables	OP (n = 304)	Control (n = 112)	*p*
Age (years)	65.20 ± 6.10	61.20 ± 5.20	0.067
Mean of age at menarche (years)	13.57 ± 1.84	13.68 ± 1.72	0.488
Mean of age at menopause (years)	45.10 ± 7.10	44.64 ± 7.14	0.422
Lumbar spine (L1-L4) (g/cm^2^)	0.626 ± 0.103	0.932 ± 0.051	<0.001 *
Total hip (g/cm^2^)	0.652 ± 0.117	1.058 ± 0.139	<0.001 *
Femoral neck (g/cm^2^)	0.540 ± 0.117	0.936 ± 0.107	<0.001 *
25-hydroxyvitamin D (ng/mL)	29.09 ± 9.24	32.26 ± 11.03	0.046 *
Calcium (mg/dL)	9.52 ± 1.20	9.52 ± 0.87	0.622
Alkaline phosphatase (U/L)	82.20 ± 48.48	90.69 ± 62.86	0.499
Gamma-glutamyltransferase (U/L)	22.45 ± 7.44	22.22 ± 7.36	0.829
Phosphorus (mg/dL)	3.50 ± 0.67	3.59 ± 0.56	0.353
Parathyroid hormone (pg/mL)	56.48 ± 27.07	59.13 ± 45.72	0.287
Creatinine clearance (ml/min)	104.00 ± 11.67	106.10 ± 10.81	0.196
Magnesium (mg/dL)	2.06 ± 0.28	2.02 ± 0.47	0.956

Demographic and clinical quantitative variables were expressed as mean and standard deviation. * indicates statistically significant.

**Table 2:** Allelic and genotypic frequencies of the *FDPS* SNV rs2297480 in the study population.

Model	Alleles/Genotypes	Controls, n (%)	OP, n (%)	OR	95% CI	*p*
	A	159 (71)	408 (67)	1		
	C	65 (29)	200 (33)	1.19	0.85–1.70	0.31
Codominant	AA	54 (48.2)	142 (47)	1		0.15
	AC	51 (45.5)	124 (41)	0.92	0.59–1.45	
	CC	7 (6.2)	38 (12)	2.06	0.87–4.90	
Dominant	AA	54 (48.2)	142 (46.7)	1		
	AC-CC	58 (51.8)	162 (53.3)	1.06	0.69–1.64	0.79
Recessive	AA-AC	105 (93.8)	266 (87.5)	1		
	CC	7 (6.2)	38 (12.5)	2.14	0.93–4.95	0.05
HWE		0.36	0.19			

OP, osteoporosis; HWE, Hardy–Weinberg equilibrium; *p*, chi-square test *p*-value; OR, odds ratio; CI, confidence interval.

**Table 3:** Association analysis of the serum levels of the biochemical markers with the genotypes for the *FDPS* SNV rs2297480.

*FDPS* (rs2297480)	25(OH)D (ng/mL)	Calcium (mg/dL)	ALP (U/L)	Phosphorus (mg/dL)	PTH (pg/mL)	Mg (mg/dL)
AA	30.74 ± 10.12	9.45 ± 0.91	82.22 ± 46.51	3.56 ± 0.54	48.34 ± 23.42	2.10 ± 0.29
AC	29.94 ± 9.62	10.22 ± 7.45	95.53 ± 59.80	3.44 ± 0.66	62.20 ± 39.18	1.96 ± 0.39
CC	27.96 ± 9.13	9.39 ± 0.92	96.70 ± 42.11	3.76 ± 0.84	76.45 ± 31.57	2.16 ± 0.45
*p*	0.523	0.726	0.020 *	0.255	<0.001 *	0.329

*FDPS*, farnesyl diphosphate synthase; 25(OH)D, 25-hydroxyvitamin D; ALP, alkaline phosphatase; PTH, parathyroid hormone. Clinical quantitative were expressed as mean and standard deviation. * indicates statistically significant.

**Table 4:** Clinical and biochemical markers of responder (OP-R) and non-responder (OP-NR) patients.

Variables	OP-R (n = 20)	OP-NR (n = 40)	*p*-Value
Age (years)	72.76 ± 6.70	69.03 ± 7.27	0.06
Age at menarche (years)	14.29 ± 2.24	13.59 ± 1.56	0.19
Age at menopause (years)	42.60 ± 8.47	45.06 ± 7.81	0.28
25-hydroxyvitamin D (ng/mL)	32.43 ± 11.71	32.17 ± 9.66	0.83
Calcium (mg/dL)	9.55 ± 0.51	9.89 ± 0.59	0.08
Alkaline phosphatase (U/L)	114.60 ± 56.81	137.80 ± 60.70	0.38
Phosphor (mg/dL)	3.49 ± 0.57	3.41 ± 0.45	0.67
Parathyroid hormone (pg/mL)	62.66 ± 32.10	57.46 ± 22.53	0.83
Magnesium (mg/dL)	1.76 ± 0.25	2.1 ± 0.20	0.028 *

Demographic and clinical quantitative variables were expressed as mean and standard deviation. * indicates statistically significant.

**Table 5:** Allelic and genotypic frequencies of *FDPS* SNV rs2297480 in non-responder (n = 18) and responder patients (n = 39) to alendronate sodium therapy.

Model	Alleles/Genotypes	OP-R, n (%)	OP-NR, n (%)	OR	95% CI	*p*
	A	24 (67)	50 (64)	1		
	C	12 (33)	28 (36)	1.12	0.45–2.85	0.83
Codominant	AA	6 (33.3)	16 (41)	1		0.08
	AC	12 (66.7)	18 (46.2)	0.56	0.17–1.85	
	CC	0 (0)	5 (12.8)	ND	0.00-ND	
Dominant	AA	6 (33.3)	16 (41)	1		
	AC-CC	12 (67.7)	23 (59)	0.72	0.22–2.32	0.58
Recessive	AA-AC	18 (100)	34 (87.2)	1		
	CC	0 (0)	5 (12.8)	ND	0.00-ND	0.045 *
HWE		0.1	1			

OP-R, osteoporosis responder; OP-NR, osteoporosis non-responder; HWE, Hardy–Weinberg equilibrium; *p*, chi-square test *p*-value; OR, odds ratio; CI, confidence interval; ND, not determined; *, statistically significant.

**Table 6:** Correlation analysis between *FDPS* gene expression and bone biochemical markers levels.

Gene	Parameters	25(OH)D	Calcium	ALP	Phosphorus	PTH	Mg
OP-R							
*FDPS*	r	−0.0831	0.3707	0.5821	−0.6954	−0.1959	0.9262
	*p*-value	0.80	0.26	0.10	0.017 *	0.56	0.023 *
OP-NR							
*FDPS*	r	−0.1408	−0.2246	0.4351	−0.0010	−0.0273	−0.4878
	*p*-value	0.61	0.36	0.15	0.99	0.92	0.26

*FDPS*, farnesyl diphosphate synthase; 25(OH)D, 25-hydroxyvitamin D; ALP, alkaline phosphatase; PTH, parathyroid hormone; Mg, magnesium. Clinical quantitative variables were expressed as (r), Pearson correlation coefficient. * indicates statistically significant.

## Data Availability

Data are contained within the article.
